# Next-generation sequencing diagnostics of bacteremia in pediatric sepsis

**DOI:** 10.1097/MD.0000000000026403

**Published:** 2021-06-25

**Authors:** Thomas Schmoch, Jens H. Westhoff, Sebastian O. Decker, Annabell Skarabis, Georg F. Hoffmann, Christian Dohna-Schwake, Ursula Felderhoff-Müser, Caroline Skolik, Manuel Feisst, Christina Klose, Thomas Bruckner, Steffen Luntz, Markus A. Weigand, Kai Sohn, Thorsten Brenner

**Affiliations:** a Department of Anesthesiology and Intensive Care Medicine, University Hospital Essen, University Duisburg-Essen, Essen; b Department of Anesthesiology, Heidelberg University Hospital; c Department of Pediatrics I, University Children's Hospital Heidelberg, Heidelberg; d Department of Pediatrics I, Neonatology, Pediatric Intensive Care, Pediatric Neurology, University Hospital Essen, University Duisburg-Essen, Essen; e Institute of Medical Biometry, University of Heidelberg; f Coordination Centre for Clinical Trials (KKS), Ruprecht-Karls-University, Heidelberg; g Fraunhofer Institute for Interfacial Engineering and Biotechnology, Stuttgart, Germany.

**Keywords:** blood culture, infants, neonates, next generation sequencing, sepsis, toddlers

## Abstract

**Introduction:**

Sepsis and septic shock are the most severe forms of infection affecting predominantly elderly people, preterm and term neonates, and young infants. Even in high-income countries sepsis causes about 8% of admissions to pediatric intensive care units (PICUs). Early diagnosis, rapid anti-infective treatment, and prompt hemodynamic stabilization are crucial for patient survival. In this context, it is essential to identify the causative pathogen as soon as possible to optimize antimicrobial treatment. To date, culture-based diagnostic procedures (e.g., blood cultures) represent the standard of care. However, they have 2 major problems: on the one hand, in the case of very small sample volumes (and thus usually in children), they are not sufficiently sensitive. On the other hand, with a time-to-result of 2 to 5 days, blood cultures need a relatively long time for the anti-infective therapy to be calculated. To overcome these problems, culture-independent molecular diagnostic procedures such as unbiased sequence analysis of circulating cell-free DNA (cfDNA) from plasma samples of septic patients by next-generation sequencing (NGS) have been tested successfully in adult septic patients. However, these results still need to be transferred to the pediatric setting.

**Methods:**

The Next GeneSiPS-Trial is a prospective, observational, non-interventional, multicenter study used to assess the diagnostic performance of an NGS-based approach for the identification of causative pathogens in (preterm and term) neonates (d1–d28, n = 50), infants (d29 to <1 yr, n = 50), and toddlers (1 yr to <5 yr, n = 50) with suspected or proven severe sepsis or septic shock (according to the pediatric sepsis definition) by the use of the quantitative sepsis indicating quantifier (SIQ) score in comparison to standard of care (culture-based) microbiological diagnostics. Potential changes in anti-infective treatment regimens based on these NGS results will be estimated retrospectively by a panel of 3 independent clinical specialists.

**Discussion:**

Neonates, infants, and young children are significantly affected by sepsis. Fast and more sensitive diagnostic approaches are urgently needed. This prospective, observational, non-interventional, multicenter study seeks to evaluate an NGS-based approach in critically ill children suffering from sepsis.

**Trial registration:**

DRKS-ID: DRKS00015705 (registered October 24, 2018). https://www.drks.de/drks_web/navigate.do?navigationId=trial.HTML&TRIAL_ID=DRKS00015705

## 1 Introduction

Sepsis and septic shock are the most severe forms of infection.^[1]^ In addition to elderly patients, sepsis affects most often young children, as well as term and, in particular, preterm neonates, causing about 8% of admissions to pediatric intensive care units (PICUs) in high-income countries.^[2–8]^ Worldwide, an estimated 1.2 million cases of childhood sepsis occur each year.^[3,8]^ Therefore, with 2202 cases per 100,000 live births, the main burden is on neonates. Moreover, beyond the newborn period, children with chronic diseases have an increased risk of developing sepsis including hematologic and oncologic diseases, metabolic, neurologic, cardiac, and renal diseases as well as solid organ transplanted children.^[9]^ Both in adults and children, the triad of early diagnosis, rapid initiation of a causal therapy (consisting of a broad anti-infective therapy with prompt source control), and immediate hemodynamic stabilization is pivotal for patient survival. While the initial antimicrobial treatment regimen has to be empirical in most cases, the identification of the causative pathogen is crucial for patient disease course. This is due to the fact that an early de-escalation of the antibiotic regime, defined as the change from a broad-spectrum antibiotic agent to one with a narrower spectrum, is associated with lower mortality in patients with sepsis or septic shock.^[10]^ However, it is only safe to de-escalate within the first 72 hours once culture-results are available. But although these culture-based methods still represent the gold standard for pathogen identification today, they are associated with considerable limitations.^[11,12]^ First of all, culture-based methods are associated with a relevant time delay. Depending on the amount of microbes as well as the particular species in the original sample, it takes up to 5 to 7 days until the final results, including resistance patterns, are available.^[13]^ Secondly, they are often false-negatives. Even in highly selected patients suffering from proven endocarditis, using 1 set of blood cultures (1 aerobic and 1 anaerobic bottle, 8–10 mL each), bacteremia or fungemia can be detected with a sensitivity of around 70%.^[14]^ Sensitivity increases to 80% to 90% with the second set of blood cultures and over 98% can be achieved with the third.^[14]^ In less preselected patients with septic shock due to various infection foci, positive rates of blood cultures were only at 11%.^[15]^ In small children, positivity rates are even lower as such volumes (6 × 10 mL = 60 mL) cannot be collected in young children (e.g., a term newborn with a bodyweight of around 3 kg has a blood volume of only about 240 mL) without resulting in severe hemodynamic instability. As a result, blood-cultures of pediatric patients with sepsis remain negative in >90% of cases,^[16]^ making a reliable de-escalation of the antibiotic treatment regime more difficult. Thirdly, culture-based methods for pathogen detection have a high risk of contamination, whereby the results become false positive. As a result, patients suffering from sepsis or septic shock are frequently over-treated with antimicrobial drugs which, in turn, favors the selection of multi-drug resistant pathogens and increases the risks of drug-related toxicities. The latter are particularly significant in preterm and term neonates, in which detoxification-systems have not yet been fully developed.^[17]^

In order to overcome these challenges, we recently introduced the concept of an unbiased sequence analyses of circulating cell-free deoxyribonucleic acid (cfDNA) by next-generation sequencing (NGS) for sepsis diagnostics.^[18–20]^ Using a quantitative score (sepsis indicating quantifier [SIQ] score), this NGS-based approach enables clinicians on the one hand to identify the relevant infecting organisms and on the other to differentiate the sepsis-causing pathogen from potential microbial contaminants (e.g., coagulase-negative staphylococci). Therefore, this method has proven to be superior to state-of-the-art molecular approaches for the diagnosis of infecting organisms (such as polymerase chain reaction [PCR]-based methods for pathogen identification) in specimens of septic patients. In contrast to these conventional PCR-based diagnostic procedures, NGS is an open approach and therefore not limited to the identification of a predefined list of probable species.

As newborns, infants, and toddlers are particularly susceptible to the toxic side effects of anti-infective drugs due to immature detoxification systems, they could exceptionally benefit from this quicker, more sensitive diagnostic tool, needing only 1 mL of patient blood.

## 2 Question and justification of the project (rationale)

This prospective, observational, non-interventional, multicenter study on the diagnostic use of NGS in neonates (d0–d28, n = 50), infants (d29 to <1 yr, n = 50), and toddlers (1 yr to <5 yrs, n = 50) suffering from severe sepsis or septic shock is designed to provide evidence for the effectiveness of an NGS-based approach in the quantitative measurement of pathogen load in blood samples from patients in the above-mentioned age groups. It is a clinical trial on a new technique designed to overcome a long-lasting problem on pediatric intensive care units and to replace the previous culture-based gold standard of microbial diagnosis in septic children. The study protocol of the Next GeneSiPS-Trial is very close to that of the Next GeneSiS-Trial, which investigated the performance of an NGS-based approach using the SIQ-Score in 500 adult patients with sepsis and septic shock.^[21,22]^ After a successful interim analysis of the Next GeneSiS-Trial, the Next GeneSiPS-Trial now seeks to extent the findings to newborns, infants, and toddlers.

## 3 Objectives

First of all, the Next GeneSiPS-Trial aims to evaluate the performance of a NGS-based approach for the detection of the relevant infecting organisms in newborns, infants, and toddlers with suspected or proven severe sepsis or septic shock by the use of the quantitative sepsis indicating quantifier (SIQ) score in comparison to standard (culture-based) microbiological diagnostics. Second, we plan to assess the clinical value of NGS-based diagnostics asking a panel of 3 independent clinical experts to retrospectively identify potential changes in patient management strategies based on the presented NGS results.

Moreover, secondary objectives will be assessed:

- Evaluation of antimicrobial resistance patterns and virulence factors.
- Evaluation of processing times for NGS-based measurements.
- Diagnostic or prognostic value of host nucleosome positioning patterns derived from plasmatic cell-free DNA in patients with suspected or proven severe sepsis or septic shock.^[23,24]^
- Diagnostic value of host expression profiles including RNA-derived biomarkers in patients with suspected or proven severe sepsis or septic shock.
- Examination of the metabolome in plasma and urine to identify risk profiles.

## 4 Trial design

The Next GeneSiPS-Trial is a prospective, observational, non-interventional, multicenter study. This study protocol follows the Standard Protocol Items: Recommendations for Interventional Trials (SPIRIT) guidelines (see Supplemental File 1).

## 5 Methods

### 5.1 Study setting

The Next GeneSiPS-Trial will be carried out as a multicenter study on pediatric and neonatal intensive care units (PICUs and NICUs) of tertiary care hospitals throughout Germany. The Department of Anesthesiology and Intensive Care Medicine, Essen University Hospital, will coordinate the study in close cooperation with the clinical studies coordination center of Heidelberg University, Heidelberg (Germany). The Institute for Medical Biometry and Informatics (IMBI), Heidelberg (Germany) is responsible for data management and will provide statistical analysis. The Fraunhofer Institute for Interfacial Engineering and Biotechnology (IGB) will provide next generation sequencing (NGS) devices and perform NGS-based microbiological diagnostics. Moreover, IGB will calculate the individual SIQ scores in plasma samples of included newborns, infants, and toddlers with severe sepsis or septic shock.

### 5.2 Eligibility criteria

Newborns (d0–d28), infants (d29 to <1 yr) and toddlers (1 yr to <5 yrs) with severe sepsis or septic shock (according to the pediatric sepsis definition) within the first 24 hours following onset (=time of diagnosis) are eligible for study inclusion. Further inclusion and exclusion criteria are described in detail in Tables 1 and 2.

**Table 1:** Inclusion and exclusion criteria of the Next GeneSiPS-Trial.

Inclusion criteria septic patients	
Age <5 years	
Written declaration of informed consent from a parent/legal guardian.	
Proven or suspected severe Sepsis (with an onset ≤24 h)SIRS (Table 3) in the presence of or as a result of suspected or proven infection.AndAcute respiratory distress syndromeOrTwo or more other organ dysfunctions as defined in Table 2.	
Or Septic shock (with an onset ≤24 h)Sepsis and cardiovascular organ dysfunction as defined in Table 2.	
Inclusion criteria control patients	
Age <5 years	
Written declaration of informed consent from a parent/legal guardian.	
Children undergoing elective surgery under general anesthesia.	
Installation of a vein access and an indwelling urine catheter required as part of the elective operation.	
Exclusion criteria	
Age ≥5 years	
Refusal to give consent	
Patient will probably be discharged from the ICU within the first 72 h following inclusion	
Palliative treatment intent	
Clinician is not committed to aggressive treatment	
Death is deemed imminent and inevitable	
Patients who had previously been included, but are readmitted to the ICU during the same hospitalization, will not be included a second time.	

**Table 2:** Organ dysfunction criteria as stated in the pediatric sepsis definitions^[23]^.

Organ system	Symptoms
Cardiovascular dysfunction	Despite the administration of isotonic intravenous fluid bolus ≥40 mL/kg in 1 h:• Decrease in BP (hypotension) <5th percentile for age or systolic BP <2 SD below normal for age^∗^OR• Need for vasoactive drug to maintain BP in normal range (dopamine >5 μg/kg/min or dobutamine, epinephrine, or norepinephrine at any dose)And• Two of the following- Unexplained metabolic acidosis: base deficit >5.0 mEq/L- Increased arterial lactate >2 times upper limit of normal- Oliguria: urine output >0.5 mL/kg/h- Prolonged capillary refill: >5 s- Core to peripheral temperature gap >3 °C
Respiratory^†^	• PaO_2_/FIO_2_ <300 mm Hg in the absence of cyanotic heart disease or preexisting lung diseaseOr• PaCO_2_ >65 torr. or 20 mm Hg over baseline PaCO_2_Or• Proven need^‡^ or >50% FIO_2_ to maintain saturation >92%Or• Need for non-elective invasive or noninvasive mechanical ventilation^§^
Neurologic	• Glasgow Coma Score ≤11^[65]^Or• Acute change in mental status with a decrease in Glasgow Coma Score ≥3 points from abnormal baseline
Hematologic	• Platelet count <80,000/mm3 or a decline of 50% in platelet count from highest value recorded over the past 3 days (for chronic hematology/oncology patients)Or• International normalized ratio >2
Renal	• Serum creatinine ≥2 times upper limit of normal for ageOr• 2-fold increase in baseline creatinine
Hepatic	• Total bilirubin ≥4 mg/dL (not applicable for newborn)Or• ALT 2 times upper limit of normal for age

Of note, pediatric and neonatal sepsis are defined slightly differently than sepsis in adults.^[23]^ While sepsis is defined in adults by the “Third International Consensus Definitions for Sepsis and Septic Shock (SEPSIS-3)” as “life threatening organ dysfunction caused by a dysregulated host response to infection”,^[1]^ the definition of pediatric sepsis is an adaptation of the previous version (SEPSIS-2),^[25]^ defining sepsis as the combination of ≥2 out of 4 criteria of a systemic inflammatory response syndrome (SIRS) (Table 3) due to an (probable) infection. “Severe sepsis” was characterized by an organ dysfunction in the context of sepsis, and “septic shock” was characterized by hypotension or the need of vasopressors despite adequate initial fluid resuscitation as a result of sepsis.^[25]^ The pediatric sepsis definitions, published 2005 by Goldstein et al,^[23]^ essentially adapt the included vital signs and laboratory variables taking into account the age-specific variations (Table 3). Comparable to SEPSIS-2, in the pediatric definition of sepsis, the term “severe sepsis” describes the combination of sepsis and organ dysfunction, which correlates closely with the definition of “sepsis” according to the “adult” SEPSIS-3 definition (Table 4). Therefore, it is not surprising that the international guidelines for the treatment of sepsis and septic shock in adults and children are very similar.^[8,11]^

**Table 3:** Age-specific SIRS criteria.

Age group	Hart rate, L/min	Respiratory rate, L/min	Leukocyte count, L/nL	Systolic blood pressure, mm Hg	Temperature, °C
0d to 1 wk	<100 or >180	>50	>34	<65	<36 or >38.5
1 wk to 1 mo	<100 or >180	>40	>19.5 or <5	<75	<36 or >38.5
1 mo to 1 yr	<100 or >180	>34	>17.5 or <5	<100	<36 or >38.5
2–5 yrs	>140	>22	>15.5 or <6	<94	
6–12 yrs	>130	>18	>13.5 or <4.5	<105	
13–18 yrs	>110	>14	>11.0 or <4.5	<117	
SIRS criteria in adults	>90	>20	>12.0 or <4.0	NA	<36 or >38.0

**Table 4:** Overview of sepsis definitions in children and adults.

	SEPSIS-1	SEPSIS-2	SEPSIS-3	Pediatric sepsis definition
Patient group	Adults	Adults	Adults	Pediatric
Authors	Bone et al^[68]^	Levy et al^[25]^	Singer et al^[1]^	Goldstein et al^[23]^
Year of publication	1992	2003	2016	2005
Sepsis	2/4 SIRS criteria	2/4 SIRS criteria or other sepsis symptoms out of a list		SIRS criteria age-adapted
Severe sepsis	Sepsis + organ dysfunction	Sepsis + organ dysfunction	Increase in SOFA-Score ≥2 points	Sepsis + organ dysfunction
Septic shock	Sepsis + hypotonia		Sepsis + hypotonia^∗^ + serum lactate ≥2 mmol/L	Sepsis + hypotonia

In addition, a total of 60 healthy children (20 neonates [d0–d28], 20 infants [d29 to <1 yr], and 20 toddlers [1 yr to <6 yr]) will be included in the study as reference to validate a pediatric SIQ-Score (pSIQ-Score).

### 5.3 Interventions

None.

### 5.4 Outcomes

The clinical performance of the NGS-based diagnostic approach in young children (newborns [d0–d28], infants [d29 to <1 yr], and toddlers [1 yr to <5 yr]) with severe sepsis or septic shock will be evaluated by a panel of 3 independent experts, who are not associated with the study site. The specialists will retrospectively interpret each case and evaluate whether the knowledge of NGS results might potentially have changed the respective treatment strategy. For this purpose, the members of the panel will receive clinical case summaries including NGS results and standard-of-care results from all samples tested. Moreover, they will be provided with all results of microbiological routine diagnostics from all further specimens (e.g., body fluid, tissue, bronchoalveolar lavage fluid [BALF], and endotracheal aspirate) that they, as treating physicians, would have had available in the 72 hour period before and after the NGS diagnosis. To identify potential changes in the antimicrobial management of patients that may have occurred if the results from the NGS-based technology had been available for clinical use, the panel will be provided with a special questionnaire, which has been adapted from the previously published Next GeneSiS-Trial.^[22]^

A secondary subgroup analysis is aimed to evaluate the clinical value of the use of NGS-based diagnostics for children suffering from a failure of empiric treatment within the first 3 days after sepsis onset (as assessed by death of the child or a lack of improvement of the child's clinical condition [in terms of an inadequate decrease of the age-adapted SOFA-score^[25]^] or persistently high procalcitonin levels).

### 5.5 Description of analysis techniques

Standard-of-care microbiological analyses: microbiological testing of potential pathogens in the different specimens will be performed according to the usual standard operating procedures of the respective participating institution.

Here, we exemplify briefly the blood culture testing at Heidelberg University Hospital which has been described in detail elsewhere^[27]^:

After the sterile collection of 0.5 to 10 mL whole blood, it is injected into an aerobic (and if possible half of it in an anaerobic) blood culture bottle (BACTEC PLUS, BD Biosciences, Heidelberg, Germany). In cases in which it is not possible to collect a sufficient amount of blood, only one aerobic blood culture bottle is inoculated. The blood culture bottles are then incubated for at least 5 days (BACTEC, BD Biosciences). Positive cultures are analyzed using VITEK2 (Biomerieux, Nuertingen) or MALDI TOF (Bruker, Madison, WI) and then subjected to an automated resistance test (VITEK 2). Herpes simplex-1 (HSV-1) or cytomegalovirus (CMV) DNA from plasma or tracheal secretions is identified by means of a quantitative real-time (RT) PCR.^[28]^ Wound swabs, catheter tips, and stool samples are also cultured using standardized methods.^[29,30]^

### 5.6 Next-generation sequencing (NGS)

The NGS-based diagnostics in the plasma samples of the study patients is carried out as follows (subject to any further technical developments).^[20]^ After the aseptic removal of 2.7 mL EDTA blood, EDTA plasma is obtained immediately by centrifugation for 10 minutes at 2500 × *g*. The plasma is then pipetted into 0.5 mL Eppendorf or 1.5 mL DNA LoBind tubes. Then, 80 μL is pipetted into a separate 0.5 mL Eppendorf tube in order to later determine the concentration of the reactive metabolite methylglyoxal (MG) from this.^[31]^ The Eppendorf tubes are then stored at –80 °C until further processing. Sample processing should be completed no later than 2 hours after the blood sample has been taken. The sample is transported for further processing to the Fraunhofer Institute for Interfacial Engineering and Biotechnology (Fraunhofer IGB, Nobelstr. 12, 70569 Stuttgart) on dry ice. Here, plasma will be centrifuged again for 10 minutes at 16,000 × *g* and 4 °C and nucleic acids will be isolated using the QIAsymphony DSP Circulating DNA Kit (Qiagen, Hilden, Germany). The isolated nucleic acids (=freely circulating DNA [cfDNA]) are then quantified using the Qubit dsDNA HS Assay Kit (Life Technologies, Carlsbad, CA). In addition, quality control is carried out using the HS NGS Fragment Analysis Kit using a Fragment Analyzer or FEMTO Pulse (Advanced Analytical Technologies Inc., Heidelberg, Germany). The so-called NGS libraries are made from 0.5 to 1 ng cfDNA using the Nextera XT Library Preparation Kit (Illumina, San Diego, CA) or NEXTflex Cell Free DNA-Seq Kit (Bioo Scientific, Austin, TX) and then sequenced on a HiSeq2500 (Illumina, San Diego, CA). In the next step, human cfDNA is identified using NextGenMap. Reads mapping to the human reference genome (hg19, minimum identity between read and reference genome of 80%) and reads with low complexity (consecutive stretches of di- and trinucleotides along the whole read sequence) are excluded from further analysis.^[32]^ Finally, reads are assigned to systematic classification using the RefSeq database (release version 68; comprising 35,749 bacterial and 4340 viral genomes complemented by 12 selected fungal genomes) using the Kraken Taxonomic Sequence Classification System (Center for Computational Biology, Johns Hopkins University, Baltimore, MD).^[33,34]^ To quantify the respective reads, read counts are normalized to the corresponding library size. Of note, *Xanthomonas* species are excluded from further analyzes as they are well-known contaminants.^[35]^

### 5.7 Sepsis indicating quantifier (SIQ)-score

The quantity of different species in microbiological samples will be assessed using the sepsis indicating quantifier (SIQ)-score.^[20]^ To calculate the SIQ-score, it is necessary to use the n × (*s* + 1) dimensional count matrix *D*, in which n is the number of control samples and *s* the number of species detected in all samples. Thus, *D*_*ij*_ defines the number of reads found in control sample *i* for species *j*. *D*_*i*,(*s* + 1)_ defines the number of reads that cannot be assigned to any species,^[22]^ whereby *D*_*i*,(*s* + 1)_ is usually much larger than the *D*_*ijs*_. Therefore, the probability of pathogen type *j* being in a control sample can be estimated using Equation (1):

(1) $${\hat{p}}_{j}=\frac{\sum_{k=1}^{n}D_{k,j}}{\sum_{k=1}^{n}D_{k,s+1}},j=1,...,s+1$$

As the concentration of bacteria, fungi, or viruses is usually low, Poisson distribution (Equation (2) for read counts of species *j*) is assumed.

(2) $$\lambda_{j}=\frac{\sum_{k=1}^{n}D_{k,j}}{\text{n}}.$$

To verify this assumption for each species, a standard chi-squared goodness of fit test is performed.^[22]^ The vector *C* = (*C*_1_, …, *C*_s_, *C*_s + 1_) results for reads in the analyzed patient plasma samples. Assuming a Poisson distribution with the species-specific parameter *λ*_*j*_, the *P*-value for at least *C*_*j*_ hits in a patient sample can be calculated according to Eq. (3).

(3) $$P(X\geq C_{j}\text{|}\lambda_{j})=\sum_{k\geq C_{j}}\frac{e^{-\lambda_{j}}\lambda_{j}^{k}}{k!}.$$

If this *P*-value is small, the hypothesis that “the number of hits of pathogen type *j* in the respective patient sample corresponds to the Poisson distribution found in healthy test subjects” must be rejected. It follows that the corresponding pathogen appears too often in the respective patient sample. With a defined species-specific *λ*, the “Sepsis Indicating Quantifier Score” (SIQ score) can now be derived (4) achieving a quantitative and probabilistic assessment of every detected microbe in the respective sample.

(4) $$SIQ_{j}=C_{j}*-(\text{lo}{\text{g}}_{10}[P(X\geq C_{j}\text{|}\lambda_{j})])$$

### 5.8 Data collection and participant timeline

Once after enrollment, basic data will be collected and, if necessary, corrected or supplemented during the course of the study. These basic data include patient demographics (age, sex, etc), relevant underlying conditions (such as oncologic or hematologic diseases, metabolic or neurological disorders, lung or kidney dysfunction), and information on pre-existing immunosuppressive diseases or the use of immunosuppressive medication for example in the context of transplanted patients.^[36]^ Moreover, the time of admission to hospital or the intensive care unit, referrer characteristics (emergency room, operating theater, recovery room, normal ward, intermediate care station), recent medical history (preoperations, reoperations, etc), suspected or proven infectious focus, and anti-infective treatment in the days and weeks preceding to the enrolment in the study will all be recorded. In addition, at sepsis onset (=onset) and 72 hours later (=72 hours): the following parameters are recorded: procalcitonin (PCT), leukocyte count, C-reactive protein (CRP), body temperature, the need for invasive ventilation, Horowitz quotient (mm Hg), oxygenation index (OI), catecholamine requirements (noradrenaline, adrenaline, dobutamine, dopamine) (μg/kg/min), 24 hours total balance (±mL/24 h), need for renal replacement therapy, age-adapted Sequential Sepsis-related Organ Failure Assessment (SOFA) and quick (q) SOFA score,^[26]^ procedures for the diagnosis/treatment of infection, radiological testing for the diagnosis/evaluation of potential infection, indwelling vascular access devices, vital status, and the antimicrobial therapy on the intensive care unit (preparation, start, and end of the respective application). Laboratory parameters will be determined in the analysis center of the respective participating institution. Discharge data will include date of discharge (ICU and hospital), discharge destination (general hospital floor, skilled nursing facility, and home), and vital status at discharge (survival/death). The final outcome evaluation of patients will be performed at 28 days after onset. A detailed flow chart of the trial specific procedures, assessments, and visits for participants is provided in Table 5.

**Table 5:** Detailed flow chart of specific procedures, assessments, and visits (Spirit figure).

Flow chart of visits	Check	Visit 1	Visit 2	Visit 3	Visit 4
Time frame		Onset		72 h	28d
Eligibility criteria	•				
Written informed consent	•				
Baseline data		•			
Clinical data		•		•	
Next generation sequencing		•		(•)	
Blood cultures		•		(•)^†^	
PCT-measurement		(•)^∗^		(•)^∗^	
Urine samples		(•)^∗^	(•)^∗^		
Outcome evaluation					•

### 5.9 Data collection from reference patients

For the 60 reference patients, only basic data will be recorded. Blood samples serve as reference values.

### 5.10 Microbiological diagnostics

Parallel to the collection of the basic data, at sepsis onset, 72 hours after sepsis onset and, if indicated by the treating physician, at a maximum of one further time within the first 3 days after sepsis onset, samples for NGS-based diagnosis with subsequent calculation of the SIQ score by the Fraunhofer IGB will be collected. Routine microbiological findings from other sample materials (e.g., surgical swabs, drainage secretions, tracheal secretions, tissue samples, etc) are included in the evaluation, provided that they have been preserved in a time window of 72 hours before or after plasma samples are obtained for NGS-based diagnostics.

### 5.11 Sample size

For reasons of feasibility, only 150 children (50 neonates [≤28 d], 50 infants [29 d to <1 year], and 50 children up to preschool age [1 year up to the age of <5 years]) will be included in this study. Due to the exploratory nature of this trial no concrete sample size calculation has been performed. However, the Next GeneSiPS-Trial is to be seen in the context of the Next GeneSiS-Trial,^[22]^ in which a total of 500 adult patients with sepsis/septic shock are included.

### 5.12 Recruitment

All neonates, infants, and toddlers up to the age of 5 years (up to the 5th birthday), who are treated on an ICU in one of the participating centers (6–10 hospitals of maximum care), who have suspected or proven severe sepsis or septic shock (according to the pediatric sepsis definition^[23]^) and whose parents or legal representatives consent to this study will be considered for study inclusion.

### 5.13 Recruitment of reference patients

Children (20 newborns [≤28 d], 20 infants [29 d to <1 year], and 20 children up to preschool age [1 year up to the age of <5]) without signs of infection undergoing elective surgery under general anesthesia requiring the installation of a central venous catheter and a urine catheter will be included as reference patients.

### 5.14 Data collection methods

Data collection will be carried out as described previously for the Next GeneSiS-Trial.^[22]^ All data collected in this trial will be recorded on electronic case report (eCRF) specifically adapted for the study using an electronic data capture (EDC) system (/REDCap, Vanderbilt University, Nashville, TN).^[37,38]^ The investigators are responsible for ensuring that all parts of the eCRFs are filled in correctly. The completed eCRF must be approved by the investigator or by a designated sub-investigator.

### 5.15 Data management

According to §13 of the Good Clinical Practice Ordinance,^[39]^ all important trial documents (e.g., eCRFs) will be archived for at least 10 years after completion of the clinical trial.

### 5.16 Statistical methods

As the Next GeneSiPS-Trial has to be understood as an extension of the Next GeneSiS-Trial, statics will be carried out as previously published.^[22]^ In brief, case by case NGS results will be compared with those obtained using standard-of-care microbiological testing. The McNemar test and Cohen *κ* statistics are used to check the agreement and concordance.^[40,41]^ All percentages and confidence intervals (Cis) are calculated using exact methods and rounded accordingly. The clinical value of NGS-based diagnostics is determined as follows: 3 independent experts (not associated with the study site) compare the results obtained using NGS-based diagnostics with those of routine microbiological diagnostics and evaluate the findings using a special questionnaire with regard to their relevance for the anti-infective therapy strategy. The evaluation of the questionnaire is carried out according to the majority rule. Further subgroup analyses will focus on patients in whom the anti-infective therapy did not work sufficiently, that is, in whom organ dysfunction did not improve or who have died (Chi-squared test for categorical data and further methods of variance analysis for continuous data). The statistical evaluation is carried out by means of SAS (SAS Institute, Cary, NC). Detailed descriptive statistics will be provided for all data collected. The chi-square test will be used for evaluating categorical variables and *t* tests will be used for evaluating continuous data univariably. Appropriate regression methods will be used for multivariable analyses. A *P*-value of <.05 is assumed to be statistically significant. However, given that the Next GeneSiPS-Trial is a not statistically powered study of exploratory nature, these values are only of descriptive value.

### 5.17 Data monitoring

Each participating site is assigned to a clinical monitor of the Coordination Centre for Clinical Trials (KKS), Heidelberg University (Heidelberg, Germany). By keeping close contact with the respective site investigators (e-mail, telephone, video call apps), the monitor will ensure that the trial is being conducted according to the protocol and regulatory requirements. If necessary, the clinical monitor will visit the participating sites to review the entries into the eCRFs on the basis of source documents.

### 5.18 Harms

Due to the non-interventional character of the Next GeneSiPS-Trial, study-related adverse events (AE) are restricted to the complications of study-related blood draws of a maximum of 3 draws of 2.7 mL within 72 hours. Blood will only be taken during routine blood draws. Therefore, it is not possible to cause additional harm by study-related vascular punctures. For the same reason, no serious AEs (SAE) (death, a life-threatening state, a prolongation of existing hospitalization, a persistent or significant disability or incapacity due to study participation) are expected. Nevertheless, possible (S)AEs can be recorded in the eCRF.

### 5.19 Auditing

No scheduled audits by the sponsor are intended. In case the competent authorities require on-site inspection or audit, the investigator must ensure the availability of all documents and support the work at any time.

### 5.20 Ethics

The study will be conducted according to standard operating procedures (SOPs) meant to ensure that all parties involved abide by the principles of Good Clinical Practice (GCP)^[39,42]^ and the Declaration of Helsinki.^[43]^ Moreover, the Next GeneSiPS-Trial will be carried out in accordance with local statutory and implementing provisions.

### 5.21 Approval of the institutional review board

Patients will not be enrolled until there is a positive vote from the institutional review board (IRB). The first IRB to approve the study design was the Ethics Committee of the Medical Faculty of Heidelberg (Trial Code No. S-605/2018).

### 5.22 Protocol amendments

Changes to the protocol have to be made in writing and require the approval of all signatories of the protocol. Subsequent amendments also require a positive assessment from the competent IRB.

### 5.23 Consent or assent

The study includes neonates, infants, and toddlers up to the age of <5 years who suffer from severe sepsis or septic shock during their inpatient stay in one of the participating study centers. A parent or legal guardian will be informed in detail about the background, content and objectives of the study in an informative discussion prior to enrollment in the study. Furthermore, the parents or legal representative will receive the information leaflet attached to this application. Written consent is required and documented by the signature of the parents or the legal representative on the declaration of consent. A child-friendly education is not suitable in this setting, as the children are very young and at the time of inclusion are severely impaired by the underlying disease. In addition, no specific cooperation by the children is required. Apart from the low blood loss, no harm to the children is foreseeable. In particular, no separate blood collection/puncture is required.

### 5.24 Confidentiality

Data collected are handled in accordance with the provisions of the General Data Protection Regulation (GDPR) of the European Union and the European Council.^[44]^ All data containing the identity of the patients or control patients are only accessible to the attending doctors, nursing staff, and clinical monitor. For all other investigators involved in the study, the data are encrypted to a pseudonym. Likewise, only pseudonyms are used in publications. Only the investigators of the respective study site are able to assign pseudonyms to patient names. Decryption of the pseudonyms is only permitted in justified cases (e.g., important new findings with regard to the diagnosis and therapy of the underlying disease). Re-identification is also permitted if a patient wishes to assert their right to information or revocation.

## 6 Discussion

In the 2016 International Guidelines for Management of Sepsis and Septic Shock (of adult patients), the Surviving Sepsis Campaign (SSC) states that “appropriate routine microbiologic cultures always include at least two sets of blood cultures (aerobic and anaerobic).” The reason for this is the fact that the first pair of blood cultures achieves only a sensitivity of about 70% regarding the identification of the causative pathogens of bloodstream infections.^[14,15]^ The second pair accomplishes a sensitivity of 80% to 90%, resulting in over 98% with the third pair.^[14,15]^ If one considers all septic patients (and not only those with bloodstream infections), the causative pathogen is only successfully identified in one-third of cases.^[45–47]^ Underlying reasons might be technical shortfalls in blood culture acquisition, locally limited foci, fastidious organisms, or very low rates of viable microorganisms in blood stream further reduced by the beginning of antibiotic treatment before sample acquisition.^[22,48]^ In children, these limitations are of particular importance as blood volumes are small. Therefore, the SSC “international guidelines for the management of septic shock and sepsis-associated organ dysfunction in children” recommends “obtaining blood cultures before initiating antimicrobial therapy” but only “in situations where this does not substantially delay antimicrobial administration,”^[8]^ as a delayed onset of anti-infective treatment increases organ dysfunction in pediatric sepsis.^[48,49]^ In this context, the SSC mentions the potential benefit of new molecular technologies including the restriction, that currently used sequencing systems are limited to a predefined set of detectable pathogens.^[8,50]^ A prominent example is the direct detection of the (deoxy)ribonucleic acid (DNA/RNA) sequences of pathogens by means of polymerase chain reaction (PCR). It is a highly sensitive and specific, culture-independent method for the targeted detection of a specific pathogen.^[28–30,51,52]^ In particular, it enables the detection of pathogens that are difficult to cultivate, such as atypical pneumonia pathogens, viruses, or fungi.^[28,51]^ A pathogen can potentially be detected even if it has already been killed or significantly reduced in number by the first administration of antimicrobial therapy.^[53,54]^ However, in this context, it is necessary to distinguish between pathogen-specific and so-called broad-spectrum or universal PCR methods. In the first case, the DNA of specific pathogens is identified by the use of specific primers, while in the second case, primers are used which bind to conserved sections of the genome of bacteria or fungi. These so-called eubacterial or pan-fungal PCRs allow a broader search approach, but are significantly less sensitive. Moreover, it is often only possible to identify the pathogen family rather than the specific species.

In contrast, NGS is able to overcome the aforementioned weaknesses of a PCR-based technique as it uses a data-driven approach without any idea of the suspected species. Therefore, no specific primer design is required and bacterial, fungal, and viral pathogens can be identified in a single assay. In a slightly modified variant, NGS is even able to correctly identify mutations of the ribonucleic acid (RNA) virus Sars-Cov-2.^[55]^ However, despite NGS becoming increasingly more important in clinical microbiology (e.g., for strain typing or microbiome studies), only sporadic reports of NGS-analyzed clinical specimens have been published to date.^[56–64]^ Moreover, in contrast to these cases, the approach used in the trial presented here also offers the possibility of quantifying the patients’ pathogen load. In addition, using the SIQ sore our approach is able to provide a statement regarding the clinical significance of the finding.^[18,20]^

The Next GeneSiPS-Trial presented here seeks to extend the results of the Next GeneSiS-Trial to newborns, infants, and toddlers up to the age of 5 years. Young children in these age groups could particularly benefit from NGS-based diagnostics, since it significantly increases the chance to correctly identify the causative pathogen, and reduces the required blood sample volume and thus reduces the stress placed on children at the same time. Moreover, as children are particularly susceptible to toxic side effects of anti-infective drugs NGS might help to decrease therapy-associated risks and side effects. Taken together, NGS has the potential to be a disruptive innovation that might replace the old gold standard of culture-based microbiological diagnostics in pediatric sepsis in the intermediate-term.

### 6.1 Justification for the enrolment of participants not capable of giving consent

In the “Next-Generation Sequencing (NGS) diagnostics of bacteremia in sepsis” (Next-GeneSiS)-Trial (DRKS-ID: DRKS00011911), we sought to validate the NGS-SIQ diagnostic approach to identify the causative pathogen in adult patients with sepsis and septic shock. The study presented here is intended to transfer this evidence to preterm and term neonates, infants, and young children. This is only possible if patients <18 years of age are enrolled.

### 6.2 Trial status

Protocol version: Version: 1.2 (December 11, 2020).

Patient and data recruitment will start in September 2021 in all study centers which have approval from their IRB. Further study centers will be added as soon the protocol has been approved by their IRB.

## Acknowledgments

The authors are indebted to the Institute of Medical Biometry and Informatics, Heidelberg and the Coordination Centre for Clinical Trials Heidelberg for ongoing support.

## Author contributions

**Conceptualization:** Thomas Schmoch, Jens H. Westhoff, Sebastian O. Decker, Annabell Skarabis, Thomas Bruckner, Steffen Luntz, Markus A. Weigand, Kai Sohn, Thorsten Brenner.

**Data curation:** Christina Klose, Caroline Skolik, Manuel Feisst.

**Formal analysis:** Caroline Skolik, Manuel Feisst.

**Funding acquisition:** Thomas Schmoch, Annabell Skarabis, Kai Sohn, Thorsten Brenner.

**Investigation:** Thomas Schmoch, Kai Sohn, Thorsten Brenner.

**Methodology:** Thomas Schmoch, Christina Klose, Kai Sohn.

**Project administration:** Thomas Schmoch.

**Resources:** Thorsten Brenner.

**Software:** Caroline Skolik, Manuel Feisst.

**Validation:** Caroline Skolik, Manuel Feisst.

**Visualization:** Thomas Schmoch.

**Writing – original draft:** Thomas Schmoch, Jens H. Westhoff, Thorsten Brenner.

**Writing – review & editing:** Thomas Schmoch, Sebastian O. Decker, Georg F. Hoffmann, Christian Dohna-Schwake, Ursula Felderhoff-Müser, Thomas Bruckner, Steffen Luntz, Markus A. Weigand, Kai Sohn, Thorsten Brenner.

## Correction

Equation 3 was originally misprinted with a line between k and j which has since been removed.

## Supplementary Material

Supplemental Digital Content
