# Supplementary figures and images for: Next-generation sequencing diagnostics of bacteremia in pediatric sepsis
Source: Medicine (Baltimore). 2021 Jun 25;100(25):e26403. doi: 10.1097/MD.0000000000026403 (PMC8238315; doi:10.1097/MD.0000000000026403)

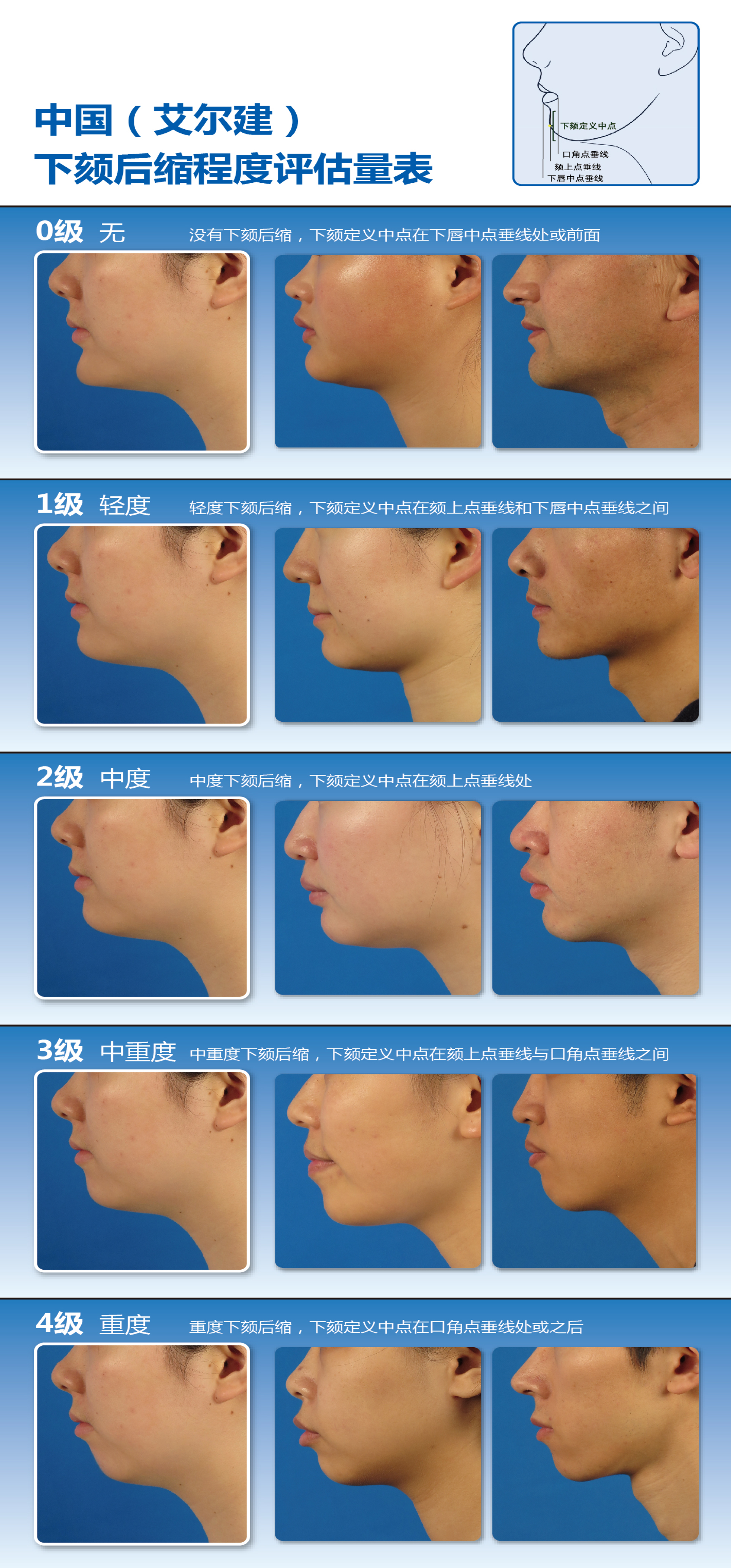

Supplement: Supplemental Digital Content [file medi-100-e26403-s001.jpg]
